# Iron Based Catalysts Used in Water Treatment Assisted by Ultrasound: A Mini Review

**DOI:** 10.3389/fchem.2018.00012

**Published:** 2018-02-02

**Authors:** Nan Zhang, Guang Xian, Xuemei Li, Panyue Zhang, Guangming Zhang, Jia Zhu

**Affiliations:** ^1^School of Construction and Environment Engineering, Shenzhen Polytechnic, Shenzhen, China; ^2^School of Environment and Natural Resource, Renmin University of China, Beijing, China; ^3^School of Environmental Science and Engineering, Beijing Forestry University, Beijing, China

**Keywords:** ultrasound, iron, sonocatalyst, organic pollutants, mechanisms

## Abstract

The characteristics and performances of catalyst are the key in catalytic ultrasonic treatment of wastewater, and iron based catalysts are known for low cost, high accessibility and safety. This paper reviewed the current research status of iron-based catalysts in water treatment assisted by ultrasound. Zero valent iron, Fe_3_O_4_ and iron composited with other metals were analyzed, their behaviors in catalytic sonochemistry were summarized, and the potential catalytic mechanisms were discussed in details. Finally, the future development in this field was proposed.

## Introduction

**Figure 1:**
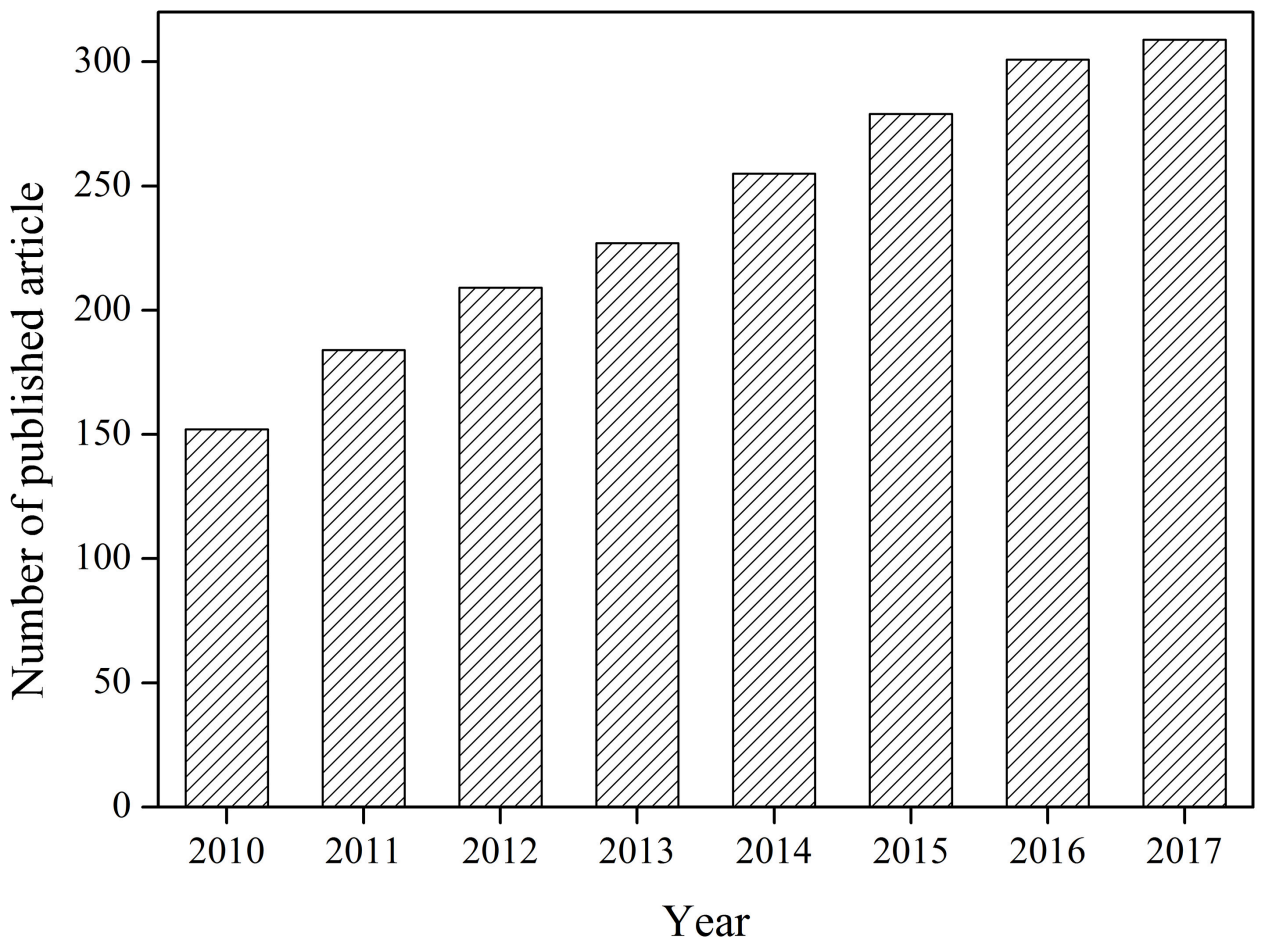
Number of articles on the subject of “catalyst and ultrasound” from 2010 to 2017.

Each year, many kinds of refractory organic pollutants are charged into water (Richardson and Kimura, 2017). These pollutants are hard to be removed by conventional treatment methods, and development of advanced treatment technology is necessary. Ultrasound has attracted great attentions for its safety, easy operation, high degradation efficiency, and free of secondary pollutants (Pokhrel et al., 2016). But removal efficiencies of organic pollutants by ultrasound are sometimes low and the energy consumption is high. Combination with catalyst to form catalytic sono-reactions can improve the efficiency of organic pollutants removal and reduce the energy consumption (Chatel et al., 2016). Many catalysts have been applied in the ultrasonic degradation of pollutants (Descorme, 2017). More and more papers are published in this field from 2010 to 2017 according to search results of the Web of Science (Figure 1).

Among all types of catalysts, iron-based catalysts are of the highest potential due to their low cost, high safety and wide-distribution. Zero valent iron (ZVI), Fe_2_(SO_4_)_3_, FeSO_4_, FeCl_3_, and FeOOH have long been used in water and wastewater treatment. Sonication alone achieved 22% diclofenac degradation while combination of FeCeOx improved the efficiency to 81% (Chong et al., 2017). Fe^0^/TiO_2_ nano-particle catalytic sonication achieved 100% removal of reactive black 5 in 20 min (Bhaumik et al., 2017).

This paper aims to provide an overview of this developing field of iron-based catalysts in water treatment assisted by ultrasound. The properties, performances, and combination with other metals of iron-based sonocatalysts were discussed. Reaction mechanisms of sonocatalytic degradation of organic pollutants were reviewed. Future development was proposed.

## Fe-based sonocatalysts

Iron element is famous for Fenton's reagent (Fe^2+^+H_2_O_2_), a classic advanced oxidation technology. However, hazards associated with the transport, handling and storage of bulk quantities of H_2_O_2_ have made the process unsafe and economically challenging. The combination of ultrasound and iron-based catalysts can achieve high degradation efficiency of organic pollutants without H_2_O_2_ (Chandran et al., 2014). Iron based sonocatalysts, ZVI, Fe_3_O_4_, and iron composited with other metals (lutetium and silver), have been examined. The reaction scheme is based on sonochemistry and Fenton-like reaction. The powerful ultrasound dissociates water to form·OH and H_2_O_2_, which then reacts with Fe^2+^ and Fe^3+^ ions in sonocatalysts as shown in Equations (1–5) (Jamalluddin and Abdullah, 2014). The catalytic activity of Fe^0^ is also based on the surface chemistry reactions of Fe^0^ to initiate the reactions in water, as shown in Equations (6–8). Application of Fe^0^ as catalyst has a few advantages: enhance mass transfer to the surface of the catalyst, continuous damage of the catalyst surface to create more defects, and cleaning of the catalyst surface (Güyer and Ince, 2011).

(1) $$\begin{array}{r} H_{2}O\left(g\right)+ultrasound\to \cdot OH\left(aq\right)\;+\cdot H\left(aq\right) \end{array}$$

(2) $$\begin{array}{r} \cdot OH\left(aq\right)+\cdot OH\left(aq\right)\to H_{2}O_{2}\left(aq\right) \end{array}$$

(3) $$\begin{array}{r} Fe^{2+}\left(aq\right)+H_{2}O_{2}\left(aq\right)\to Fe^{3+}\left(aq\right)+\cdot OH\left(aq\right)+OH^{-}\left(aq\right) \end{array}$$

(4) $$\begin{array}{r} Fe^{2+}\left(aq\right)+\cdot OH\left(aq\right)\to Fe^{3+}\left(aq\right)+OH^{-}\left(aq\right) \end{array}$$

(5) $$\begin{array}{r} Fe^{3+}\left(aq\right)+H_{2}O_{2}\left(aq\right)\to Fe^{2+}\left(aq\right)+\cdot OOH\left(aq\right)+H^{+}\left(aq\right) \end{array}$$

(6) $$\begin{array}{r} Fe^{0}\left(s\right)+ultrasound\to Fe^{2+}\left(aq\right)+2e^{-} \end{array}$$

(7) $$\begin{array}{r} Fe^{0}\left(s\right)+2Fe^{3+}\left(aq\right)\to 3Fe^{2+}\left(aq\right) \end{array}$$

(8) $$\begin{array}{r} Fe^{0}\left(s\right)+H_{2}O_{2}\left(aq\right)\to Fe^{2+}\left(aq\right)+\cdot OH\left(aq\right)+OH^{-}\left(aq\right) \end{array}$$

Table 1 summarizes the typical iron-based sonocatalysts. The dechlorination of Fe^0^ for complicated chlorinated compounds is often incomplete and with low efficiency. Luo et al. (2010) found that the Ag/Fe catalyst was quite effective for the degradation of chlorinated organics, and silver also provide some disinfection effect.

**Table 1:** Ultrasonic catalytic degradation of organic pollutants by iron sonocatalysts.

	**Sonocatalyst**	**Organic pollutant**	**Removal efficiency (%)**	**References**
1	Reactive ZVI	Diclofenac	k = 0.0786 min^−1^	Güyer and Ince, 2011
2	Fe^0^	Phenol	90 (TOC)	Segura et al., 2012
3	ZVI aggregate	C.I. Direct Red 23	95	Weng and Tsai, 2016
4	Rectorite-supported nanoscale ZVI	Methyl orange and metronidazole	93 and 97	Yuan et al., 2016
5	Chitosan-stabilized nanoscale ZVI	Acid fuchsine	99	Jin et al., 2016
6	Fe_3_O_4_ nanoparticles	2-hydroxyethyl cellulose	k = 3.9 × 10^10^ mol^−1^·L^−1^·min	Taghizadeh and Seifi-Aghjekohal, 2015
7	Nanosized Fe_3_O_4_-loaded coffee waste hydrochar	Acid red 17	100	Khataee et al., 2017
8	Fe_3_O_4_/Polyaniline	Methyl orange	100	Wang et al., 2015
9	Fe_3_O_4_-SiO_2_-TiO_2_	Ibuprofen	70	Kang et al., 2015
10	α-Fe_2_O_3_ nanoparticles	Eosin Y	72.5	Gobouri, 2016
10	Fe_2_O_3_/SBA-15	Phenolic aqueous	100	Bremner et al., 2009
11	LuFeO_3_	RhB	82.9	Zhou et al., 2015
		Acid orange 7	89	
12	Fe/Ti-NaY	Amaranth	75	Alwash et al., 2013
13	Fe-doped zeolite Y	Acid red B	100	Jamalluddin and Abdullah, 2014
14	FeCeO_*x*_	Diclofenac	83	Chong et al., 2017
15	TiO_2_ and Fe^2+^	17α-ethynylestradiol	100	Frontistis and Mantzavinos, 2012
16	Fe-TiO_2_ nanotubes	Rhodamine	99	Pang and Abdullah, 2012
17	Fe^3+^ doped TiO_2_ nanotubes	Real textile waste water	79.9	Pang and Abdullah, 2013
18	Fe-fullerene/TiO_2_	Acid red 17	92	Meng and Oh, 2011
19	Fe-based catalysts	Ibuprofen	100	Ziylan and Ince, 2015
20	Fe^2+^	Reactive blue 181	93.5	Basturk and Karatas, 2014
21	Modified montmorillonite	Acid red 17	82	Acisli et al., 2016
22	Iron–silver bimetallic nanoparticles	Tetrabromobisphenol A	100	Luo et al., 2010

When nonmagnetic catalysts (e.g., TiO_2_) are employed, their recovery is a troublesome issue. Super-paramagnetic Fe_3_O_4_ facilitates fast recovery/re-dispersion of the catalyst by simply switching on/off an external magnet (Richardson and Kimura, 2017), which is the most effective and simplest method to enhance the recovery and reuse of catalysts (Kang et al., 2015). Taghizadeh and Seifi-Aghjekohal (2015) found that the sonocatalytic activity of Fe_3_O_4_ was the best among Fe_3_O_4_, Rutile-TiO_2_, ZnO, and Anatase-TiO_2_ nanoparticles. The proposed reason was that Fe_3_O_4_ enhanced the ·OH radical generation by the electron transfer between iron ions and H_2_O molecules.

The activity of ZVI and Fe_3_O_4_ is promoted in acidic solution. However, the reuse property is poor due to severe iron leaching in acidic condition. Besides, acidic pH limits the practical application of sonocatalysts. Therefore, iron composites catalysts have been proposed. Zhou et al. (2015) found that the pH value had a small effect on the sonocatalytic degradation of RhB by LuFeO_3_, and the LuFeO_3_ particles exhibited a good structural stability with no structural change before and after sonication. Alwash et al. (2013) found that Fe/Ti-NaY kept its high activity in the decolorization of amaranth after three times of reuse; X-ray diffraction proved that the catalyst was stable after reuse and maintained its crystallinity. Another method to improve the stability of catalyst is doping the active component on supports like graphene and zeolite (Rakmae et al., 2016). Even at neutral and alkaline medium, a good decolorization and degradation efficiency could still be achieved in the present of Fe-doped zeolite Y under ultrasound. Fe (III)/Y demonstrated good catalytic efficiency, low Fe leaching, and good reusability (Jamalluddin and Abdullah, 2014).

Iron has been used to modify TiO_2_ and formed new sonocatalysts. TiO_2_ nano-tubes possess a relatively wide energy band gap (3.2 or 3.0 eV in Anatase or Rutile phase) and fast recombination rate of charge carriers (Richardson and Kimura, 2017). One possible solution to this problem is introducing suitable transition metals such as Fe, Cr, or Co into TiO_2_ to form a new sonocatalyst with narrower band gap and longer lifetime of charge carriers (Pang and Abdullah, 2013).

## Potential mechanisms of sonocatalytic degradation of organic pollutants

**Figure 2:**
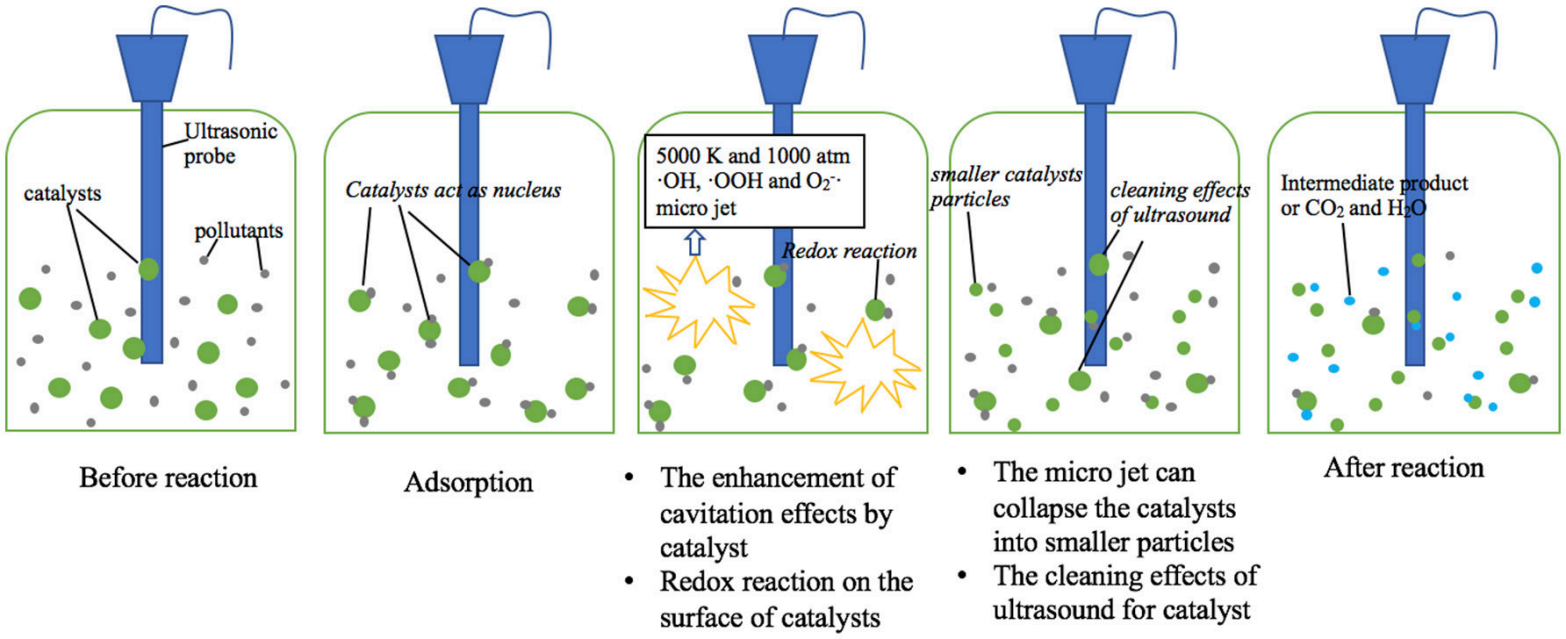
Potential mechanisms of sonocatalytic degradation of organic pollutants.

The main mechanism of ultrasound is cavitation effects, which can be enhanced by addition of sonocatalysts. Some organic pollutants are adsorbed on the surface of catalysts, thus increases the removal rate due to shorter reaction path. Meanwhile, ultrasound enhances the redox reaction between catalyst and organic pollutants. Potential mechanisms of sonocatalytic degradation of organic pollutants are shown in Figure 2.

### Cavitation effects

The most import sonocatalytic mechanism lays in the cavitation of water during ultrasonic irradiation (Wang et al., 2015). The cavitation involves the processes of formation (nucleation), rapid growth (expansion), and violent collapse (implosion) of cavitation bubbles. Such a violate collapse of cavitation bubbles cause local high temperature and pressure (up to 5,000 K and 1,000 atm) and the emission of light (sono-luminescence), and generate free radicals including ·OH, ·OOH, and ${\text{O}}_{2}^{-}\cdot$ (Pokhrel et al., 2016). The free radicals can react with target contaminants.

Mechanical effect is a main advantage of ultrasound irradiation (Alwash et al., 2013). Ultrasound can promote the mass transfer, which leads the contact of pollutants with free radical or the catalyst more sufficiently. In addition, the micro jet can collapse the catalyst into smaller particles, which offer a higher surface area, thus enhances the surface reaction.

### The enhancement of cavitation effects by catalyst

Catalyst particles in water can act as nucleus for cavitation bubbles (Zhao et al., 2014). Once the catalyst particles sizes are in the same order of magnitude with the size of the cavitation bubbles, catalyst particles can form extra nucleus of cavitation bubbles. Extra nucleus generates more cavitation bubbles, causes stronger cavitation effects, leading to higher degradation efficiency.

### Adsorption of catalysts

Organic pollutants can be adsorbed on the surface of catalysts due to electrostatic attraction and sonication (Thangavel et al., 2015). The adsorbed pollutants then react with the sono-generated free radicals from/around the catalyst. Moreover, the concentration of free radicals is high on the surface of catalysts and the adherence of organic pollutants upon the catalyst can shorten the path for radicals/cavities to decompose the pollutants. Then the degradation rate of organic pollutants is improved.

### Redox reaction between sonocatalyst and organic pollutants

Organic pollutants can react with the surface of sonocatalyst. The free radicals produced by ultrasound improve the redox reaction between sonocatalyst and organic pollutants, which accelerates the removal of organic pollutant. Free radicals react with polyvalent metal sonocatalyst such as iron-based composites, which may produce some new valence state sonocatalyst to accelerate the reaction (Chong et al., 2017).

### The cleaning effects of ultrasound for catalyst

The cleaning effect of ultrasound for catalysts refers to the phenomenon that ultrasound waves in the ultrasonic system continuously remove the intermediates or by-products from the surface of catalysts to reactive the surface (Alwash et al., 2013). The cleaning effects of ultrasound are based on the cavitation effects. Ultrasound generates many bubbles in water and they collapse fast, then the catalysts are cleaned by the shock wave. Such an ultrasonic regeneration of the sonocatalyst is of great advantage via removing the contaminants and decomposition of toxic organic pollutants (Wang et al., 2015).

## Conclusions and perspectives

In this mini-review, the typical iron-based sonocatalysts are summarized. Their sonocatalytic activity is different for different organic pollutants. The special property for iron-based sonocatalysts is magnetism, which is beneficial to separate catalysts from water. The mechanisms of sonocatalytic degradation of organic pollutants involve both ultrasound irradiation and sonocatalyst. In the future, more works will be done on this developing field and following issues might be of great values:

1. Produce better sonocatalysts
2. Obviously the sonocatalytic efficiency depends on the type of catalyst. Development of novel catalyst with higher efficiency has always been the hot topic, and better understanding of the sonocatalytic mechanisms is also necessary to promote the application of the sonocatalytic technology.
3. Pay more attention to the safety and cost efficiency
4. Most researchers only investigate the activity of sonocatalyst, but pay little attention on the economy and safety of sonocatalyst. If the materials for preparing sonocatalyst are rare or expensive and the preparation method is complex, these sonocatalysts are difficult to be applied. The stability and reusability of sonocatalyst also need be considered, which are relative to the service life of sonocatalyst.
5. Enhance the selectivity of sonocatalysts
6. In some situations, trace toxic substances and high concentration of non-poisonous pollutants co-exist in water, the sonocatalyst must have good selectivity, but little investigation is available on this subject, which needs be addressed.

## Author contributions

NZ collected and read papers and contributed partially to the Introduction and mainly to Table 1; GX collected and read papers and contributed to paper writing; XL contributed greatly to the writing of Potential Mechanisms of Sonocatalytic Degradation of Organic Pollutants section; JZ contributed to the paper design and refine; PZ contributed to paper design and data analysis; GZ was in charge of the whole writing.

### Conflict of interest statement

The authors declare that the research was conducted in the absence of any commercial or financial relationships that could be construed as a potential conflict of interest.
